# Ileocaecal Volvulus With an Intestinal Rotational Abnormality and Internal Hernia in a Paediatric Patient: A Case Report

**DOI:** 10.1155/cris/9951709

**Published:** 2024-11-25

**Authors:** Venla Soini, Matias Hilska, Marko Sallisalmi, Risto Juusela, Ella Virkki, Arimatias Raitio

**Affiliations:** ^1^Department of Surgery, Vaasa Central Hospital, Wellbeing Services County of Ostrobothnia, Vaasa, Finland; ^2^Department of Paediatric Surgery, University of Turku and Turku University Hospital, Turku, Finland; ^3^Department of Anesthesiology and Intensive Care, Vaasa Central Hospital, Wellbeing Services County of Ostrobothnia, Vaasa, Finland

**Keywords:** ileocaecal volvulus, internal hernia, intestinal rotational abnormality, malrotation

## Abstract

**Background:** Caecal volvulus in the paediatric population is uncommon, yet at worst this condition is a life-threatening surgical emergency. In children, caecal volvulus can be associated with a variety of predisposing factors such as chronic constipation, intestinal malrotation, or neurological disease.

**Case Representation:** We present a rare case of caecal volvulus, internal hernia, and an intestinal rotational abnormality in a previously healthy 8-year-old boy. The patient presented with a history of abdominal pain and vomiting for 3 days and was admitted to the hospital in a severe septic shock. After the initial stabilisation with fluids and vasopressors, an emergency laparotomy was performed. A necrotic caecum volvulus, a transmesocolic hernia, and an abnormal rotation of the small intestine were diagnosed. The necrotic bowel segment was resected in a right-sided hemicolectomy, after which a resection distal to medial colic artery was cut-off to achieve normal anatomy. The patient was discharged on the 12th postoperative day in good health and has since returned to normal active life without any health issues within the follow-up of 5 months.

**Conclusions:** Caecal volvulus and internal hernia can lead to a life-threatening condition requiring immediate surgical treatment. Rare causes of abdominal pain in children should be kept in mind when severe symptoms are present.

## 1. Background

Caecal volvulus is a condition in which the caecum twists around itself. The condition usually affects older women, and paediatric caecal volvulus is extremely rare. The literature concerning paediatric caecal volvulus consists of sporadic case reports and small case series. Most of the reports concerning paediatric caecal volvuli describe a congenital band or malfixation behind the condition such as weakness or lack of retroperitoneal attachment [1, 2].

Paediatric caecal volvulus has been linked to congenital syndromes, such as Cornelia de Lange syndrome [3, 4] and Trisomy 18 [5]. Also, Hirschsprung's disease, chronic constipation, and malrotation have been identified as predisposing factors for caecal volvulus [5–7]. Colonic volvulus in children seems to occur most often in the caecum, with reports indicating that 35%–75% of all large intestine volvuli are located in the caecum [7, 8].

The acute treatment of caecal volvulus requires surgical intervention. The operative technique depends on the general condition of the patient and is based on expert opinion rather than general guidelines due to the rarity of the condition. In literature, conservative treatment, reduction with caecopexy, and caecostomy have been associated with good results. However, permanent treatment is only achieved by intestine resection and anastomosis [6, 8].

Internal hernia refers to the passage of viscera through an internal anatomical or pathological opening in the abdominal cavity. In case of transmesocolic hernias, the hernial opening is in the mesocolon. There is typically no hernia sac, but an opening in the mesocolon. Only a few cases of transmesocolic hernias have been described in paediatric population [9–11].

## 2. Case Presentation

### 2.1. Initial Evaluation

An 8-year-old male (weight: 26 kg and height: 140 cm) with no previous medical history was admitted to hospital with history of abdominal cramps and vomiting for 3 days. He had not been able to defaecate since the start of symptoms. The out-of-hospital emergency medical services were dispatched after the patient had lost consciousness in his family's car on his way to the emergency department (ED) of a nearby surgical hospital treating both children and adults. Fluid resuscitation with isotonic crystalloid fluids was commenced by first responders, and the patient was rapidly transferred to the ED.

At the ED, the patient was clinically diagnosed to have a septic shock (HR: 149 BPM, BP: 78/52 mmHg, temperature: 39°C, and SpO_2_: 97%) with peritonitis; the patient was tachycardic, hypotensive, febrile, and had cold periphery and generalised peritonism. Abdominal ultrasound examination raised suspicion of massive gastric dilation with a small amount of free fluid in the hepatorenal recess. The first laboratory sampling showed hypokalemic, hyponatremic, and hypoglycaemic lactic acidosis with increased haematocrit and inflammatory markers (Table 1). Intravenous broad-spectrum antibiotics (Cefuroxime 30 mg/kg and metronidazole 6 mg/kg) were administered immediately after obtaining bacterial blood cultures, and the patient was urgently transferred to the operating theatre for an emergency laparotomy.

### 2.2. Anaesthetic Management

Prior to the induction of anaesthesia, and to avoid hemodynamic collapse, the ongoing hemodynamic optimisation with crystalloid fluids was augmented with infusions of norepinephrine and epinephrine (0.04–0.06 μg/kg/h) as indicated by a point-of-care echocardiographic assessment (dyskinetic ventricular septum and dyssynchronized left ventricular function) [12]. Subsequently, a rapid sequence induction of anaesthesia with intravenous propofol 0.75 mg/kg, esketamine 1.25 mg/kg and rocuronium 1.5 mg/kg was performed while maintaining cricoid pressure until the airway was secured with an endotracheal tube. Immediately after the release of cricoid pressure the patient vomited nonbilious dark emesis profusely, but not aspirate gastric contents. Anaesthesia was maintained with intravenous infusions of propofol and remifentanil.

### 2.3. Surgical Management

**Figure 1:**
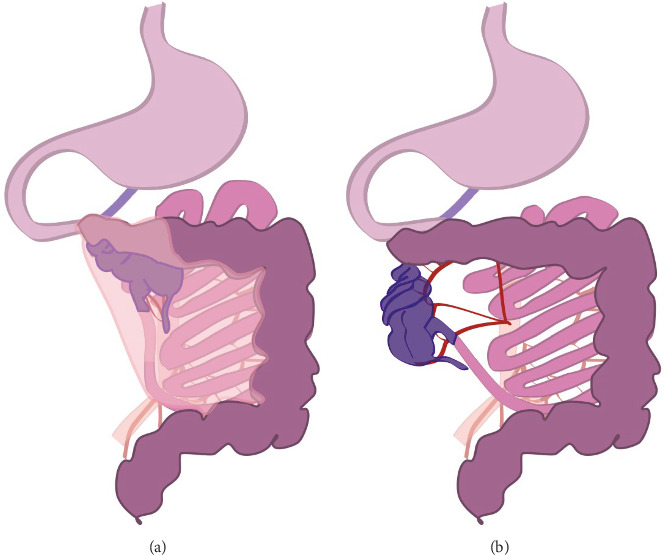
(A) Initial anatomic situation at the beginning of laparotomy, hernia sac overlapping caecum, part of transversal colon, and small intestine. Some of the intestine located in the omental bursa. (B) Anatomical situation of the right hemicolon, ceacal volvulus rotated already 90° for clarity, and low medial colic artery preventing the reposition of the small intestine.

The patient was considered too unstable for laparoscopic surgery, and the surgical team proceeded directly to an emergent midline exploratory laparotomy. Intra-abdominally, a large hernia sac, assumedly mesocolic origin, was first observed containing most of the intestine (Figure 1A). After excision of the hernia sac, extremely dilated sections of the small intestine were observed. Finally, a necrotic caecal volvulus was found atypically in the right upper abdomen, partly behind the stomach (Figure 1B). The observed 270° clockwise rotation was immediately derotated, but the volvulated intestine section could not be saved. No signs of perforation were observed.

The patient had a mesenteric hernia through the mesentery of the transverse colon. The small intestine and the right hemicolon were protruding behind the stomach. The hernia sac was bordered anteriorly by the anterior leaflet of the transverse mesocolon. The omental bursa was anatomically in the correct position, and part of the small bowel was in the bursa. Dorsally the pancreas and other structures were in their correct positions and the ligament of Treitz was also in its correct position. After reviewing the anatomical situation, a decision was made to resect the necrotic right hemicolon. The intestines were resected on both sides with Tristapler cartridges (Covidien GIA) and the mesentery was cut with a Ligasure impact instrument. In pathological analysis of the resected hemicolon findings compatible with ischaemic colitis were found.

**Figure 2:**
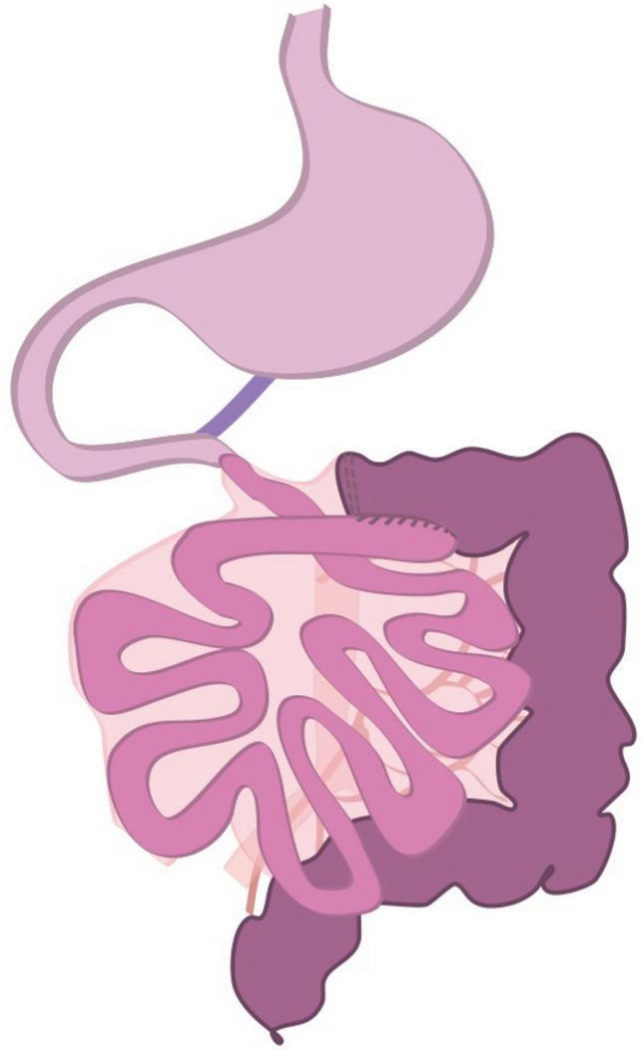
Primary anastomosis after right sided hemicolectomy.

After bowel resection, the omentum was resected from the transverse colon. However, medial colic artery pedicle prevented the small intestine from repositioning to its normal anatomy, and an additional resection with the pedicle was performed. Transverse colon was then passed to the cranial side of the small intestine and the small intestines to the right side of the patient's abdomen. The roof of the hernia sac was completely cut excised. Finally, an ileum—transversum side-to-side isoperistaltic anastomosis was performed with Tristapler cartridges, and all staple lines were embedded with hand sutures using monofilament thread (Figure 2). The mesocolon window was also closed with monofilament sutures. The laparotomy incision was directly closed in layers and a single nonsuction drainage was left in the pelvic area.

Postoperatively, a tertiary care centre with a paediatric intensive care unit and an on-call paediatric surgeon was contacted. The patient was transferred to the tertiary care centre in a mechanical ventilator and requiring hemodynamic support. In the paediatric intensive care unit, the patient was extubated, and the nonsuction drainage was removed on the first postoperative day. Epidural catheter was administered for pain management. The patient was transferred to a paediatric surgical ward on 4th postoperative day. Parenteral nutrition was administered until nasogastric tube could be removed on the 10th postoperative day. On the 12th postoperative day, the patient was discharged.

At the 2-month follow-up appointment, the patient was doing well. The wound had healed, and the abdomen was benign in palpation without any tenderness. The patient had returned to school, and active life including recreational sports. Intestinal function had returned to normal. After that there have been no contacts to health care and no complications have occurred in 10-month follow-up.

## 3. Discussion

We describe a case of an 8-year-old male with the combination of internal hernia and intestinal rotational abnormality leading to an acute caecal volvulus. We could not find any other literature reviews or case reports concerning a similar combination of caecal volvulus, internal hernia, and vascular anatomy in paediatric patients. However, cases with congenital malrotation, internal hernia, and acute midgut volvulus have been described [9, 10].

The pathophysiology of intestinal malrotation is lack of normal 270° counterclockwise rotation of the intestine around the superior mesenteric artery, which happens during fetal development. This possesses a risk of obstruction due to strangulation and volvulus [13, 14]. Our interpretation of the anatomic situation in our patient to have an intestinal rotational abnormality is based on the facts that the primary anatomic location of caecum was at right upper abdomen and the rotation of especially the right haemicolon was incomplete, which further lead to caecum nonfixation. There was no duodenal malrotation, as the ligamentum Treitz was at its correct anatomical position. The majority of jejunum was on the left side of midline. Also, despite the resection of the necrotic caecum, the intestine could not be repositioned to their normal anatomy before the resection of the middle colic artery pedicle, as the mesentery root of the small intestine was located cranially and to the left in relation to the middle colic artery pedicle. Similar anatomical situation has not been described in the literature. However, abnormality concerning the root of mesentery has been described previously, as Nahle et al. [15] stated in their report on midgut volvulus. Xiong et al. [16] described a classification system for malrotation, yet our patient's situation does not directly fit to any of these classes, and we decided to use the term intestinal rotational abnormality instead.

Brezler, Sico, and Seifarth [9] and Merrot et al. [10] have presented case reports with intestinal malrotation, transmesocolic hernia, and midgut volvulus. Classical midgut volvulus occurs in the small intestine, which makes our case unique in comparison to these previous reports. Although the acute condition differed in Brezler, Sico, and Seifarth [9] case, the patient's situation was clinically quite similar with acute abdominal pain and septic shock. However, their patient suffered a complete midgut volvulus herniated through a transmesocolic defect eventually leading to 12 operations, complete loss of the small bowel, and partial large bowel. In the case of Merrot et al. [10], no resection of the intestine was needed. The exact age of the patient was not described in the work of Brezler, Sico, and Seifarth [9], but the report suggests that the patient was considerably younger than our patient. The patient in the report of Merrot et al. [10] was 4 years old. According to previous literature, up to 80% of intestinal malrotation cases are diagnosed during infancy [17–19].

In our patient's case, the condition was eventually managed in a single operation. The solution of a direct intestinal anastomosis was courageous but proved to be worthwhile in the end. The specialists in our centre argued for this solution because the intestine was not perforated, blood flow after the resection of the necrotic segment was faultless, and the abdomen could be closed without tension. What is more, in our case only a small part of the small intestine was involved in the necrotic volvulus. Previous literature also supports the conduct of primary anastomosis in paediatric volvuli [20, 21]. Furthermore, paediatric intra-abdominal surgery has been associated with a high risk of adhesions in comparison to adults [22] and stoma formation would have increased this risk. Stapled anastomosis has shown good results also in paediatric surgery and was chosen for our patient [23]. Of course, the situation must be assessed individually in each case.

In our case a computed tomography (CT) scan was initially considered in the ED, but due to the extremely unstable condition of the patient this was not performed. The clinical situation and ultrasound imaging findings indicated a demand for emergency laparotomy. On a case reported by Brezler, Sico, and Seifarth [9], a CT scan was performed, and this showed a dilated large intestine, suspicion for ischaemic small intestine injury, and discontinuation of the superior mesenteric artery. This differs from our case as in our patient, the superior mesenteric artery was intact. If our patient had been stable at the outset, the decision to operate would have been preceded by a CT scan. However, the preoperative CT scan in our patient's case would probably not have changed the choice of treatment, as an emergency laparotomy would have been performed even if the CT scan had revealed the necrotic caecum volvulus with the anatomical situation as described. When the patient's condition is critical, the need and safety for time-consuming imaging must also be viewed critically. What is more, ultrasound imaging has been proven to provide excellent specificity in paediatric midgut malrotation and volvulus situations, even though in our patient ultrasound did not offer direct diagnosis [15, 24–26]. Also, other reports speculate, that ileocaecal artery and vein twist could be visualised in colour doppler ultrasound [5, 27]. In our opinion, a point of care ultrasound is an option to be considered when evaluating a child with abdominal pain in an emergency room.

In the beginning of symptoms, the family themselves had assumed that the situation was gastroenteritis or similar. Certainly, gastroenteritis as a differential diagnostic for abdominal pain and vomiting is much more common than caecal volvulus. Yet, if vomiting persists, and especially if the symptoms do not include diarrhoea, less common causes of abdominal pain should also be considered. Quality of emesis might also lead to diagnosis as bilious emesis is generally a symptom of intestinal obstruction and nonbilious emesis is associates with other conditions [28]. Although caecal volvulus in paediatric population is extremely rare, the complications such as caecal gangrene and perforation are severe and occur in up to 50% of the cases [5]. Overall mortality has been estimated around 10% [29, 30].

## 4. Conclusions

We believe that our unusual case of caecal volvulus, internal hernia, and abnormal anatomy adds to the existing literature concerning paediatric caecal volvulus and internal hernia situations. We underline the potential severity of the caecal volvulus, especially in the paediatric population, where the rarity of the condition may cause diagnostic delay. Clinical symptoms with point-of-care ultrasound may be sufficient to provide indication for surgery in an acute situation. A direct intestinal anastomosis is an option for the repair also in the situation of acutely ill paediatric patients.

## Figures and Tables

**Table 1:** Laboratory test results at ED.

Test	Result	Normal range
vB-pH	7.23	7.23–7.42
vB-glucose	2.2	4.0–6.0 mmol/l
vB-potassium	5.2	3.3–4.8 mmol/l
vB-sodium	131	137–144 mmol/l
vB-lactate	6.0	0.5–2.2 mmol/l
Leukocytes	14.5	4.5–13.5 10 E 9/l
Haemoglobin	183	116–154 g/l
C-reactive protein	252	<10 mg/l
Creatinine	108	33–59 µmol/l

Abbreviations: ED, emergency department; vB, venous blood gas analysis.

## Data Availability

Data will be made available upon reasonable request.

## Nomenclature

CT: computer tomography
MAP: mean arterial pressure.
